# Supplementary material for: Fatal Human H3N8 Influenza Virus has a Moderate Pandemic Risk
Source: bioRxiv. 2025 Oct 2:2025.10.02.679960. Preprint. [Version 1] doi: 10.1101/2025.10.02.679960 (PMC12621962; doi:10.1101/2025.10.02.679960)
Supplement: 1 [file NIHPP2025.10.02.679960V1-supplement-1.pdf]

bioRxiv preprint doi: https://doi.org/10.1101/2025.10.02.679960; this version posted October 2, 2025. The copyright holder for this preprint (which was not certified by peer review) is the author/funder, who has granted bioRxiv a license to display the preprint in perpetuity. It is made available under aCC-BY 4.0 International license.

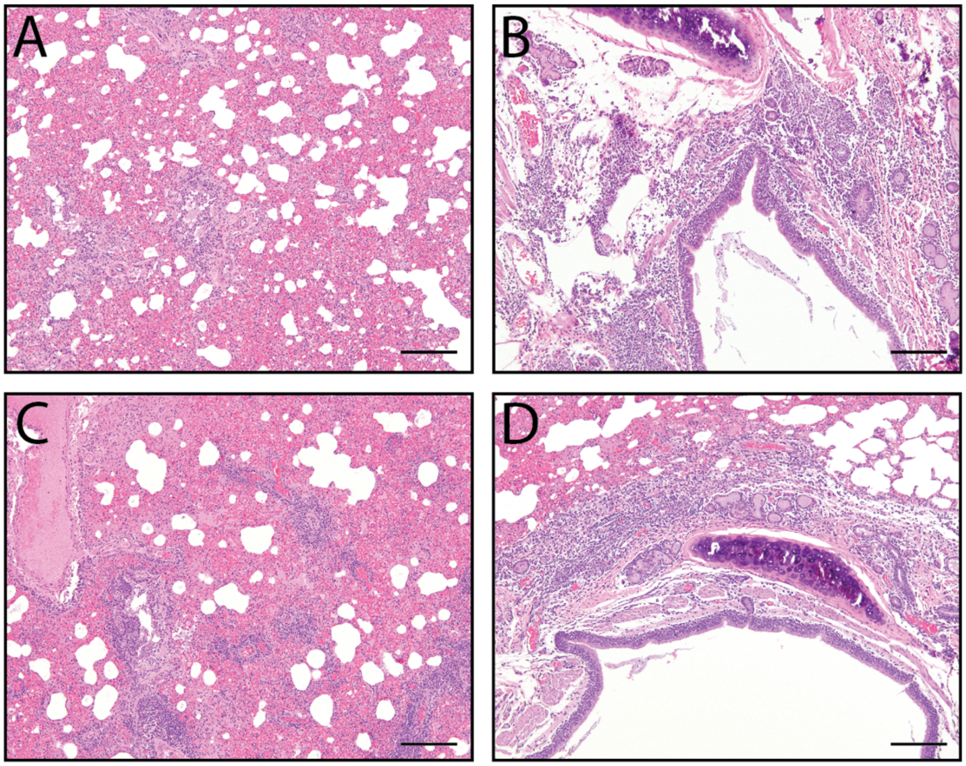

Supplemental Figure 1. Representative lung images from A/GD/F005/23 H3N8 infected ferrets at day 5 post-infection. **A, B** are from ferret without pre-existing immunity and **C, D** are from ferrets with H3N2 pre-existing immunity. Scale bar 200 μm.

bioRxiv preprint doi: https://doi.org/10.1101/2025.10.02.679960; this version posted October 2, 2025. The copyright holder for this preprint (which was not certified by peer review) is the author/funder, who has granted bioRxiv a license to display the preprint in perpetuity. It is made available under aCC-BY 4.0 International license.

	A/Guangdong/ZS-23SF005/2023	A/Henan/4-10/2022	A/Changsha/1000/2022
A/Guangdong/ZS-23SF005/2023	-	***HA*:** 360A, 275R, 285K, 557E, 277Q, 204T, 363V, 245G, 50V, 466R, 506V, 400L, 541F<br>***PB1***: 48Q, 90S, 213S, 636G, 87G, 40G, 89T, 667V, 33P, 37Q, 3Q, 172N, 525V, 738E, 374A, 42C, 1M, 339I<br>***PB2***: 444I, 661T, 184M, 64I, 627E, 299R, 118Y, 153D<br>***PA***: 432I, 647S, 272T<br>***MP***: 50F, 55I<br>***NP***: 136M<br>***NA***: 8T, 39D, 389R, 341K, 46N, 27V, 37P, 17L, 42N, V432I, N647S, N272T<br>***NS***: 48T, 79L, 75K, 205N, 59S, 76A, 212S, 127N, 72E, 124M, 60S, 73S	***HA***: 360A, 275R, 557E, 277Q, 204T, 363V, 245G, 50V, 466R, 506V, 41I, 294I, 505D<br>***PB1***: 525V, 90S, 384A, 213S, 636G, 375S, 87G, 40G, 26R, 89T, 23D, 79R, 757K, 21K, 171M, 52K, 37Q, 59K, 642N, 363K, 34N, 75R, 11Q, 83F, 5Q, 14V<br>***PB2***: 661T, 444I, 355K, 184M, 676V, 702R, 64I, 409S, 627E, 570M, 61K, 340R, 473M<br>***PA***: 432I, 647S, 343S, 272T, 203K, 387V, 626K, 337T, 465I, 626R<br>***MP***: 10L, 89S, 50F, 12K, 46I, 55I, 42M, 13T, 28V, 20S<br>***NP:*** 408V, 194I, 373A<br>***NA***: 8T, 39D, 389R, 341K, 78E, 83M, 6K, 191I, 12S<br>***NS***: 48T, 79L, 75K, 205N, 59S, 221E, 47E, 205S, 127N, 124M, 80S, 44R, 226V
A/Henan/4-10/2022	***HA***: 360E, 275K, 285R, K557, 277R, 204N, 363I, 245R, 50I, 466K, 506I, 400V, F541L<br>***PB1***: 48R, 90N, 213N, 636E, 87E, 40D, 89I, 667I, 33L, 37P, 3R, 172N, 525I, 738K, 374E, 42Y, 1T, 339V<br>***PB2***: 627K, 299K, 118F, 153V, 444V, 661A, 184T, 64M<br>***PA*:** 432V, 647N, 272N<br>***MP:*** 50C, 55F<br>***NP:*** 136L<br>***NA***: 46K, 27A, 37L, 17S, 42S, 8I, 39N, 389K, 341N,<br>***NS***: 76T, 212P, 127T, 72D, 124V, 60R, 73T, 48A, 79M, 75E, 205S, 59R	-	***HA***: 400V, 541L, 41I, 294I, 505D, 285R<br>***PB1***: 33L, 37P, 525V, 3R, 384A, 172N, 525I, 375S, 738K, 374E, 26R, 42Y, 1T, 23D, 339V, 79R, 757K, 48R, 21K, 171M, 52K, 37Q, 59K, 642N, 363K, 34N, 75R, 11Q, 83F, 5Q, 14V, 667I<br>***PB2***: 153V, 627K, 355K, 676V, 702R, 299K, 118F, 409S, 570M, 61K, 340R, 473M<br>***PA***: 343S, 203K, 387V, 626K, 337T, 465I, 626R<br>***MP***: 10L, 89S, 12K, 46I, 42M, 13T, 28V, 20S<br>***NP:*** 136L, 408V, 194I, 373A<br>***NA***: 46K, 27A, 37L, 17S, 42S, 78E, 83M, 6K, 191I, 12S<br>***NS***: 76T, 212P, 127T, 72D, 60R, 73T, 221E, 47E, 205S, 127N, 80S, 44R, 226V
A/Changsha/1000/2022	***HA***: 41V, 294V, 505N, 360E, 275K, 557K, 277R, 204N, 363I, 245R, 50I, 466K, 506I<br>***PB1***: 79L, 757N, 21M, 171I, 52N, 37R, 59R, 642S, 363R, 34S, 75H, 11R, 83S, 5R, 14A, 525I, 90S, 384S, 213N, 636E, 375N, 87E, 40D, 26Q, 89I, 23G<br>***PB2***: 627V, 570I, 61R, 340K, 473V, 661A, 444V, 355R, 184T, 676M, 702K, 64M, 409C<br>***PA***: 203R, 387I, 626R, 337A, 465V, 626K, 432V, 647N, 343A, 272N<br>***MP***: 13I, 28I, 20N, 10P, 89G, 50C, 12R, 46L, 55F, 42I<br>***NP***: 408I, 194V, 373T<br>***NA***: 78G, 83I, 6R, 191V, 12T, 8I, 39N, 389K, 341N<br>***NS***: 221K, 47D, 205R, 127D, 124V, 80N, 44K, 226I, 48A, 79M, 75E, 205S, 59R	***HA***: 41V, 294V, 505N, 285K, 400L, 541F<br>***PB1***: 79L, 757N, 48Q, 21M, 171I, 52N, 37R, 59R, 642S, 363R, 34S, 75H, 11R, F83S, 5R, 14A, 667I, 33P, 37Q, 525I, 3Q, 384S, 172D, 525V, 375N, 738E, 374A, 26Q, 42C, 1M, 23G, 339I<br>***PB2***: 627V, 570I, 61R, 340K, 473V, 153D, 627E, 355R, 676M, 702K, 299R, 118Y, 409C<br>***PA***: 203R, 387I, 626R, 337A, 465V, 626K, 343A<br>***MP***: 13I, 28I, 20N, 10P, 89G, 12R, 46L, 42I<br>***NP***: 136M, 408I, 194V, 373T<br>***NA***: 78G, 83I, 6R, 191V, 12T, 46N, 27V, 37P, 17L, 42N<br>***NS***: 221K, 47D, 205R, 127D, 80N, 44K, 226I, 76A, 212S, 127N, 72E, 60S, 73S	-

Supplemental Table 1. A pairwise comparison for the three human H3N8 samples show they differ by multiple nonsynonymous amino acid (AA) substitutions across their genomes. The table compares the amino acid differences between each pair of isolates, where each row represents one human isolate. Each cell represents the pairwise comparison between that isolate and the isolate in the corresponding column. The identities of each amino acid represent the amino acid encoded for the isolate in each row. For example, A/Henan differs from A/Guangdong at 2 amino acid sites in MP: 50 and 55. A/Henan encodes a C at 50 and an F at 55, while A/Guangdong encodes an F at 50 and an I at 55.
